# Antihyperglycemic action of rhodiola-aqeous extract in type1-like diabetic rats

**DOI:** 10.1186/1472-6882-14-20

**Published:** 2014-01-13

**Authors:** Chiang-Shan Niu, Li-Jen Chen, Ho-Shan Niu

**Affiliations:** 1 Department of Nursing, Tzu Chi College of Technology, Hualien City 97005, Taiwan; 2 Institute of Basic Medical Sciences, College of Medicine, National Cheng Kung University, Tainan City 70101, Taiwan

**Keywords:** Adrenalectomy, Hyperglycemia, Opioid μ-receptor, Plasma glucose, Rhodiola-water extract

## Abstract

**Background:**

*Rhodiola rosea* (Rhodiola) is a plant in the Crassulaceae family that grows in cold regions of the world. It is mainly used in clinics as an adaptogen. Recently, it has been mentioned that Rhodiola increases plasma β-endorphin to lower blood pressure. Thus, the present study aims to investigate the antidiabetic action of Rhodiola in relation to opioids in streptozotocin-induced diabetic rats (STZ-diabetic rats).

**Methods:**

In the present study, the plasma glucose was analyzed with glucose oxidase method, and the determination of plasma β-endorphin was carried out using a commercially available enzyme-linked immunosorbent assay. The adrenalectomy of STZ-diabetic rats was used to evaluate the role of β-endorphin. In addition, quantitative reverse transcription-polymerase chain reaction (qRT-PCR) and western blotting analysis were performed to investigate mRNA and protein expressions.

**Results:**

Rhodiola-water extract dose-dependently lowered the plasma glucose in STZ-diabetic rats and this action was reversed by blockade of opioid μ-receptors using cyprodime. An increase of plasma β-endorphin by rhodiola-water extract was also observed in same manner. The plasma glucose lowering action of rhodiola-water extract was attenuated in bilateral adrenalectomized rats. In addition, continuous administration of rhodiola-water extract for 3 days in STZ-diabetic rats resulted in an increased expression of glucose transporter subtype 4 (GLUT 4) in skeletal muscle and a marked reduction of phosphoenolpyruvate carboxykinase (PEPCK) expression in liver. These effects were also reversed by blockade of opioid μ-receptors.

**Conclusions:**

Taken together, rhodiola-water extract improves hyperglycemia via an increase of β-endorphin secretion from adrenal gland to activate opioid μ-receptors in STZ-diabetic rats.

## Background

Diabetes mainly results from dysfunction of glucose homeostasis and causes many complications, including cardiovascular diseases, neurological complication, chronic renal failure, and diabetic retinopathy [1-4]. Hyperglycemia is correlated with an increased risk of short-term acute complications, such as hyperosmolar coma, and long-term complications affecting the micro and macrovasculature [5,6]. In general, oral administration of antidiabetic agents, caloric restrictions, regular exercise and lifestyle are often applied to control hyperglycemia [7-9]. Recently, the management of diabetic hyperglycemia has attracted much attention in alternative therapy [10,11]. Moreover, herbal supplements and other alternative medicine for handling of diabetic hyperglycemia are necessary.

*Rhodiola rosea* (Rhodiola) belongs to Crassulaceae family that is grown at high altitudes and northern latitudes and this herb is mentioned to reduce fatigue, improve physical activity and alleviate depression [12,13]. The documented hydrophilic extracts from Rhodiola contain many chemical compositions and they have been identified, including cinnamic alcohol, chlorogenic acid, rhodiooctanoside, rosiridin, rosavin and the phenolic compounds salidroside, rhodiolin in addition to a novel compound consisting of viridoside with an attached arabinose unit (mongrhoside) [13,14]. In recent years, rhodiola-water extract has been reported to improve hypertension via the release of β-endorphin in animal model [15]. Otherwise, involvement of β-endorphin or endogenous opioids in glucose lowering action of natural products has been mentioned [16,17]. Actually, some chemical agents or exogenous β-endorphin might improve hyperglycemia through an activation of opioid μ-receptors in STZ-diabetic rats lacking insulin [18-20]. However, antihyperglycemic action of rhodiola-water extract remained obscure. In the present study, rhodiola-water extract is used to screen the effect on hyperglycemia in diabetic rats. Also, the mediation of β-endorphin in this action of rhodiola-water extract is further identified.

## Methods

### Plant materials

The radix of *Rhodiola Rosea* (Crassulaceae) originated from Qinghai-Tibet plateau of China was donated from Giu Ding Biotechnology Co., Ltd., Taiwan and was authenticated by Professor I-Min Liu (Department of Pharmacy, Tajen University). The dried voucher specimen (No. GiuDing 94001) was deposited in the herbarium of the college of pharmacy, Tajen University (Pingtung, Taiwan).

### Preparation of plant extracts

The air-dried chopped plant radix (150 g) was extracted exhaustively by maceration with in distilled water (1 L) by stirring (Harmony Hot Plate Stirrer, Japan) at 55°C for 7 h (three times) and The extracts were sieved using a muslin cloth and then filtered under suction pressure with a filter paper. They were then concentrated under reduced pressure at 50°C using a rotary evaporator (Buchi, Switzerland). The crude water extract was then dried under vacuum at 50°C to yield the water-soluble fraction (WtF, 16.5 g). The water-soluble fraction (WtF) was stored in a capped container and maintained at 4°C. This product was obtained from Professor Shorong-Shii Liou (College of Pharmacy, Tajen University, Pingtung, Taiwan). The major active principles quantified in this product were salidroside (8.4 mg/g) and p-tyrosol (1.9 mg/g) as described in previous reports [21,22].

### Animal models

Ten-week-old male Wistar rats weighing 250 to 300 g were obtained from the Animal Center of National Cheng Kung University Medical College. The diet of the animals used for the study was standard laboratory diet. The number of animals for each group of experiment is eight. STZ-diabetic rats were induced by intravenous injection (i.v.) of STZ (65 mg/kg) into Wistar rats according to the previous method [23]. Animals were considered to be type-1 diabetes- like model if they showed plasma glucose concentrations of 20 mM or greater in addition to polyuria and other diabetic features according to previous reports [24-30]. All studies were carried out 2 weeks after the injection of STZ. The rats used in the present study were maintained in accordance with the Guide for the Care and Use of Laboratory Animals of the National Institutes of Health, as well as the guidelines of the Animal Welfare Act and the study was approved by the animal research ethics committee of Tzu Chi College of Technology (TCCN-101006) which consisted of Prof. Yang SC, Chen MS, Dai KF, Liu WT, Wei TK, Cho LG, and Wu MY.

### Laboratory determinations

The determination of plasma glucose was conducted according to the previous study [31]. The concentration of plasma glucose was measured by the glucose oxidase method using an analyzer (Quik-Lab, Ames; Miles Inc., Elkhart, IN, USA). The determination of BER in samples was carried out using a commercially available enzyme-linked immunosorbent assay (Peninsula Laboratories, Belmont, CA, USA).

### Effect on plasma β-endorphin level in STZ-diabetic rats

After fasting overnight, STZ-diabetic rats received an intraperitoneal treatment of rhodiola-water extract at the desired doses. It has been documented that rats receiving sodium pentobarbital showed no significant change in the parameters measured in plasma [32]. Thus, animals were anesthetized with sodium pentobarbital (35 mg/kg i.p.) and blood samples (0.1 mL) were coμlected from the femoral vein for measurement of plasma glucose concentrations and β-endorphin (BER). In the present study, rhodiola-water extract at 75 mg/kg was found to produce the maximal plasma glucose-lowering effect in STZ-diabetic rats 60 min after intraperitoneal injection. Thus, the effect of rhodiola-water extract on plasma BER was determined using blood samples collected at 60 min after the treatment. STZ-diabetic rats that received an intraperitoneal treatment of vehicle only (0.9% saline) were used as controls. Further experiments were performed with the pretreatment of inhibitors, such as the antagonists of opioid μ-receptors cyprodime (1.0 mg/kg) purchased from (Tocris Cookson, Bristol, UK). The antagonist was injected intravenously into rats 30 min before the treatment of rhodiola-water extract.

### Adrenalectomy of STZ-diabetic rats

Bilateral adrenalectomy was performed using the dorsal approach under pentobarbital anesthesia (30 mg/kg, i.p.) as described previously [28]. Wistar rats to be adrenalectomized were fed standard rat chow and 0.9% sodium chloride in their drinking water ad libitum prior to surgery. Wistar rats to receive a sham operation (controls) were fed standard rat chow and water ad libitum prior to surgery. Animals were allowed to recover for 2 weeks after the operations. The animals appeared alert and in good health. Following recovery, diabetes was induced by an injection of STZ as described above. The effect of rhodiola-water extract at 75 mg/kg was determined using blood samples collected at 60 min after the treatment.

### Quantitative reverse transcription-polymerase chain reaction (qRT-PCR)

Total RNA was extracted from liver and soleus muscle tissue samples using Trizol reagent (Invitrogen). Two microgram of total RNA was used for the reverse transcription reaction, along with Superscriptase II (Invitrogen), oligo-dT, and random primers. The web-based assay design software from the Universal Probe Library Assay Design Center was used to design the TaqMan primer pairs and to select the appropriate hybridization probes. The reactions were performed in 20 μL of a mixture consisting of 13.4 μL of PCR buffer, 0.2 μL of each probe (20 μmol/L), 4 μL of LightCycler TaqMan (Roche Diagnostics GmbH) and 2 μL of template cDNA. A LightCycler Detection System (Roche Applied Science, Germany) was used for amplification and detection. The PCR reaction was carried out as follows: one cycle of 95°C for 10 min, 45 cycles of 94°C for 10 s, 60°C for 20 s, and 72°C for 1 s. The crossing point for each amplification curve was determined using the second derivative maximum method. The concentration of each gene was calculated with the aid of the LightCycler software using the respective standard curve as reference. Relative gene expression was expressed as a ratio of the target gene concentration to the housekeeping gene 36B4 concentration. The sequences of primers were forward 5′-tttctgttggtatgcataatttgtaat-3′ and reverse 5′-ccagtagaggaggtcaacaacc-3′ for GLUT 4 and forward 5′-gatgacattgcctggatgaa-3′and reverse 5′-acccgttttctgggttgatg-3′ for PEPCK.

### Western blotting analysis

Western blotting analysis was carried out as previously described [28] and quantification was obtained from three individual experiments. After homogenization of liver and soleus muscle using a glass/Teflon homogenizer, the homogenates (50 μg) were separated by sodium dodecyl sulfate-polyacrylamide gel electrophoresis, and Western blot analysis was performed using either an anti-rat GLUT 4 antibody purchased from (Abcam, Cambridge, U.K) in soleus muscle or an anti-rat PEPCK antibody from (Santa Cruz Biotechnology, CA) in liver. The blots were probed with a goat polyclonal actin antibody from (Millipore, Billerica, MA, USA) to ensure that the amount of protein loaded into each lane of the gel was constant. Blots were incubated with the appropriate peroxidase-conjugated secondary antibodies. After removal of the secondary antibodies, the blots were washed and developed using the ECL-Western blotting system. Densities of the obtained immunoblots at 45 KDa for GLUT 4, 69.5 KDa for PEPCK, and 43 KDa for actin were quantified using laser densitometer.

### Statistical analysis

The plasma glucose-lowering activity of rhodiola-water extract was calculated as the percentage decrease of the initial glucose value according to the formula: (Gi − Gt)/Gi × 100%, where Gi is the initial glucose concentration and Gt is the plasma glucose concentration after treatment of rhodiola-water extract. Data are expressed as the mean ± S.E.M. for the number (*n*) of animals in the group as indicated in tables and figures. Differences among groups were analyzed by one-way ANOVA. The Dunnett range post hoc comparisons were used to determine the source of significant differences where appropriate. A *p*-value of 0.05 or less was considered statistically significant.

## Results

### Effects of rhodiola-water extract on plasma glucose concentration and plasma β-endorphin-like immunoreactivity (BER) level in STZ-diabetic rats

**Figure 1:**
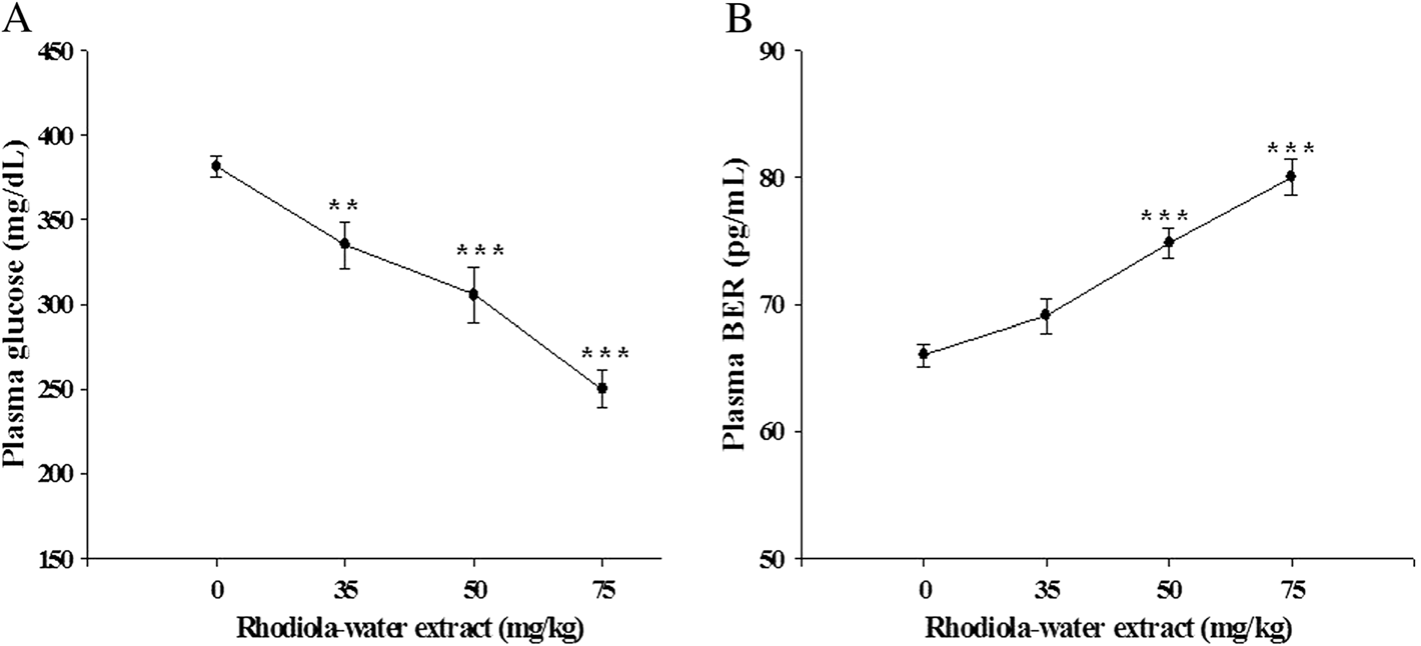
**Effects of rhodiola-water extract on plasma glucose concentration and plasma β-endorphin level in STZ-diabetic rats. (A)** The plasma glucose lowering activity produced by rhodiola-water extract through an intraperitoneal (i.p.) injection into STZ-diabetic rats. **(B)** Plasma β-endorphin-like immunoreactivity (BER) level in STZ-diabetic rats receiving i.p. injection of rhodiola-water extract. Values of mean and bar of S.E.M. were obtained from each group of 8 rats. Vehicle only (0.9% saline) was given at the same volume. **p < 0.01 and ***, *p* < 0.001 versus data from animals treated with vehicle (0).

STZ-diabetic rats treated with rhodiola-water extract at 50 mg/kg through intraperitoneal injection produced the plasma glucose-lowering activities as 8.3 ± 1.2% at 30 min later, 22.1 ± 0.8% at 60 min later, 18.7 ± 1.4% at 90 min later and 6.7 ± 2.1% at 120 min later (*n* = 8). Then, we applied it at the most effective of time point in following experiments. After 60 minutes, rhodiola-water extract shows the plasma glucose-lowering activities as 11.6 ± 1.1%, 21.1 ± 0.9%, 32.9 ± 3.2% in STZ-diabetic rats after an intraperitoneal (i.p.) injection at 35 mg/kg, 50 mg/kg or 75 mg/kg, respectively (*n* = 8). Rhodiola-water extract at 75 mg/kg significantly lowered the plasma glucose concentration from 381.4 ± 5.7 mg/dL to 250.1 ± 11.2 mg/dL (*p* < 0.001; *n* = 8) as shown in Figure 1A. Also, a dose-dependent increase of plasma BER level was observed in parallel in STZ-diabetic rats receiving rhodiola-water extract at same dosing from 35 mg/kg to 75 mg/kg (Figure 1B). Rhodiola-water extract at 75 mg/kg increased the plasma BER level from basal level of 66.0 ± 0.9 pg/mL to 80.0 ± 1.4 pg/mL in STZ-diabetic rats.

### Effect of opioid μ-receptor blockade on rhodiola-water extract-induced plasma glucose lowering action in STZ-diabetic rats

**Figure 2:**
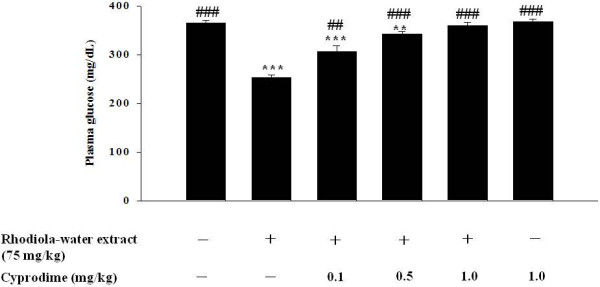
**Effect of opioid μ-receptor blockade on rhodiola-water extract-induced plasma glucose lowering action in STZ-diabetic rats.** The antagonist was given by an intravenous (i.v.) injection at 30 min before the injection of rhodiola-water extract. Vehicle (0.9% NaCl in distilled water) was given at same volume. Values (mean ± S.E.M.) were obtained from each group of 8 rats. **, *p* < 0.01 and ***, *p* < 0.001 compared with data from vehicle-treated STZ-diabetic rats. ##, *p* < 0.01 and ###, *p* < 0.001 compared with data from rhodiola-water extract (75 mg/kg)-treated STZ-diabetic rats.

As shown in Figure 2, the inhibitory effect of cyprodime on the plasma glucose-lowering activity of rhodiola-water extract in STZ-diabetic rats was produced in a dose-dependent manner. In the presence of 1 mg/kg cyprodime, the plasma glucose concentration in rats treated with 75 mg/kg rhodiola-water extract was 358.8 ± 7.6 mg/dL, which was not statistically different with the vehicle-treated STZ-diabetic rats (365.1 ± 5.5 mg/dL). However, cyprodime alone did not affect basal plasma glucose levels in STZ-diabetic rats.

### Bilateral adrenalectomy of STZ-diabetic rats abolishes the effects of rhodiola-water extract on plasma glucose and BER levels

Bilateral adrenalectomy was performed in STZ-diabetic rats. Two weeks after adrenalectomy, there were no significant differences in the basal plasma levels of glucose and BER between sham-operated and adrenalectomized STZ-diabetic rats (Table 1). However, the actions of rhodiola-water extract regarding the lowering of plasma glucose and the elevation of plasma BER levels were abolished by bilateral adrenalectomy in STZ-diabetic rats while both were unchanged in the sham-operated STZ-diabetic rats (Table 1).

**Table 1:** Effect of adrenalectomy on the rhodiola-water extract-induced changes of β-endorphin-like immunoreactivity (BER) and plasma glucose concentration in STZ-diabetic rats

	**Sham-operated group**	**Adrenalectomized group**
Plasma glucose (mg/dL)		
Basal	378.6 ± 5.3	375.0 ± 4.6
Vehicle	370.5 ± 6.9	366.5 ± 5.0
Rhodiola-water extract (75 mg/kg)	254.0 ± 2.4***	373.5 ± 5.8
Plasma BER (pg/mL)		
Basal	70.5 ± 2.1	69.8 ± 1.2
Vehicle	68.5 ± 1.8	69.0 ± 1.4
Rhodiola-water extract (75 mg/kg)	84.3 ± 1.3***	68.7 ± 1.2

Blood samples from STZ-diabetic rats receiving an intraperitoneal injection of rhodiola-water extract or a vehicle-treated control were used for determination of β-endorphin-like immunoreactivity (BER) and the plasma glucose concentration. Vehicle (0.9% saline) was given at the same volume. Values (mean ± S.E.M.) were obtained from each group of eight rats. Basal level shows the value from fasted animals without treatment. ***p < 0.001 versus basal value in each group.

### Effect of opioid μ-receptor blockade on rhodiola-water extract-induced changes of GLUT 4 in skeletal muscle and hepatic PEPCK in STZ-diabetic rats

**Figure 3:**
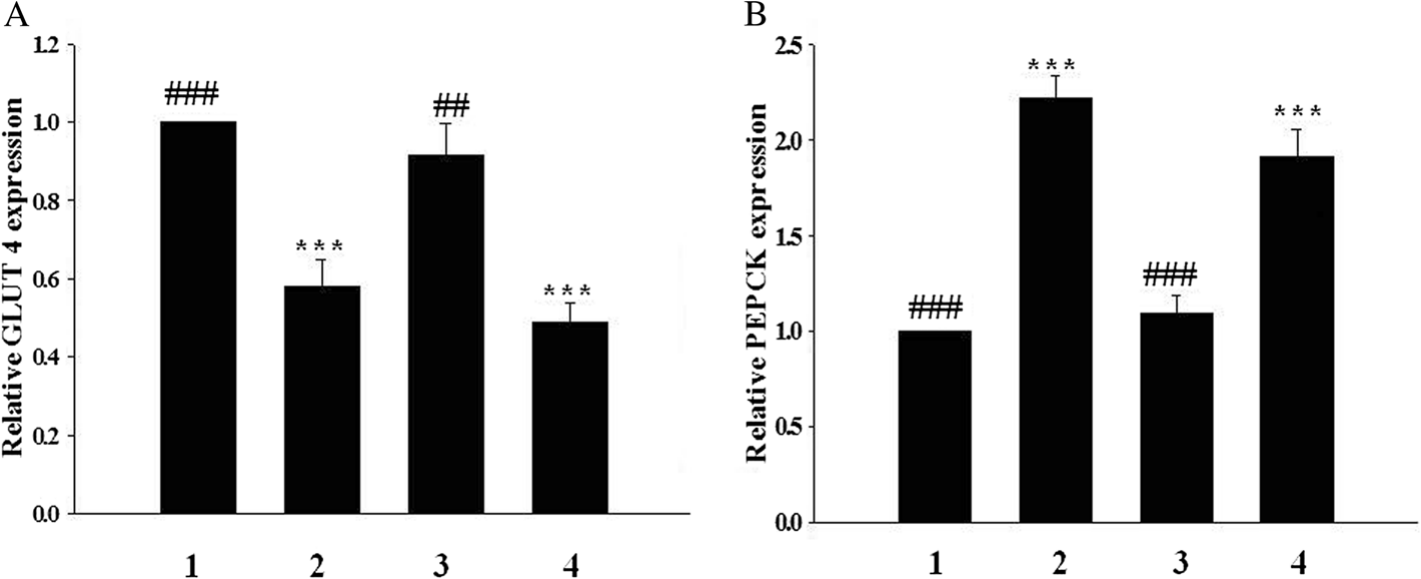
**Effect of opioid μ-receptor blockade on rhodiola-water extract-induced changes of GLUT 4 in skeletal muscle and hepatic PEPCK in STZ-diabetic rats. (A)** The mRNA level of GLUT 4 in skeletal muscle isolated from STZ-diabetic rats receiving treatment with rhodiola-water extract only or with cyprodime in combination, three times daily for 3 days. **(B)** The mRNA level of PEPCK expression in liver isolated from STZ-diabetic rats receiving same treatment with rhodiola-water extract only or with cyprodime in combination. The samples were then collected for qRT-PCR analysis. Lane1, vehicle-treated Wistar rats; lane2, vehicle-treated STZ-diabetic rats; lane 3, rhodiola-water extract (75 mg/kg)-treated STZ-diabetic rats; lane 4, rhodiola-water extract (75 mg/kg) plus cyprodime (1.0 mg/kg)-treated STZ-diabetic rats. Data are expressed as mean with standard error (SE) (n = 6 per group) is indicated in each column. ***, *p* < 0.001 compared with data obtained from lane 1. ##, *p* < 0.01 and ###, *p* < 0.001 compared with data obtained from lane 2.

Treatment of STZ-diabetic rats with rhodiola-water extract (75 mg/kg) three times daily for 3 days resulted in an elevation of GLUT 4 expression level in skeletal muscle and this action was attenuated by cyprodime (1.0 mg/kg) (Figure 3A). In addition, the expression level of PEPCK in liver of STZ-diabetic rats was increased to about 2.2 folds of that in non-diabetic rats. The reduction of PEPCK expression by rhodiola-water extract (75 mg/kg) in diabetic rats was observed and this action was also attenuated by cyprodime (Figure 3B).

### Effect of opioid μ-receptor blockade on rhodiola-water extract-induced changes of protein levels of GLUT 4 in skeletal muscle and hepatic PEPCK in STZ-diabetic rats

**Figure 4:**
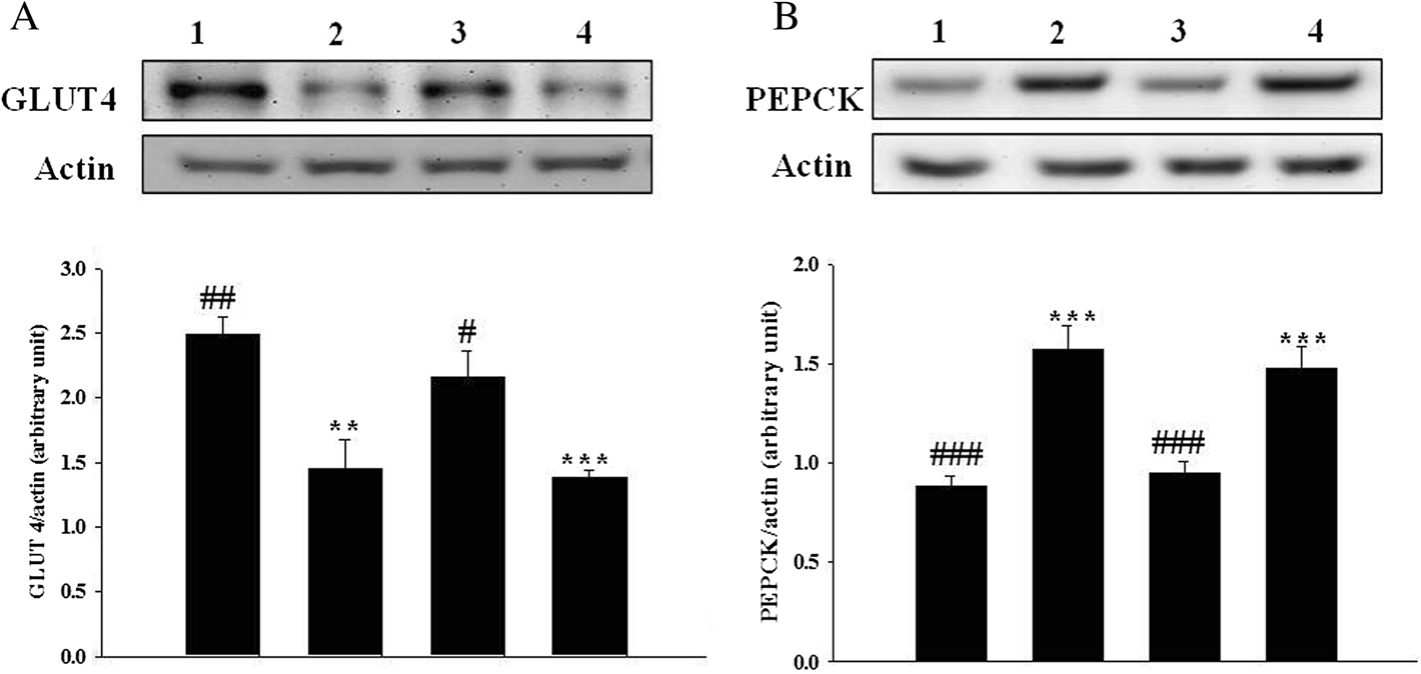
**Effect of opioid μ-receptor blockade on rhodiola-water extract-induced changes of protein levels of GLUT 4 in skeletal muscle and hepatic PEPCK in STZ-diabetic rats. (A)** The representative response of protein level for GLUT 4 or actin in skeletal muscle isolated from STZ-diabetic rats receiving treatment with rhodiola-water extract only or with cyprodime in combination, three times daily for 3 days. **(B)** The representative response of protein level for PEPCK or actin in liver isolated from STZ-diabetic rats receiving same treatment with rhodiola-water extract only or with cyprodime in combination. Lane1, vehicle-treated Wistar rats; lane2, vehicle-treated STZ-diabetic rats; lane 3, rhodiola-water extract (75 mg/kg)-treated STZ-diabetic rats; lane 4, rhodiola-water extract (75 mg/kg) plus cyprodime (1.0 mg/kg)-treated STZ-diabetic rats. Quantification of protein level using GLUT 4/actin or PEPCK/actin showing mean with standard error (SE) (n = 6 per group) in each column is indicated in the lower panel. **, *p* < 0.01 and ***, *p* < 0.001 compared with data obtained from lane 1. #, *p* < 0.05, ##, *p* < 0.01 and ###, *p* < 0.001 compared with data obtained from lane 2.

In Western blotting analysis, continuous treatment with rhodiola-water extract (75 mg/kg) increased the GLUT 4 protein level in skeletal muscle. Pretreatment of STZ-diabetic rats with cyprodime (1.0 mg/kg) abolished this action of rhodiola-water extract (Figure 4A). Similarly, the protein level of PEPCK in liver of STZ-diabetic rats was raised to approximately 1.8 folds of that in non-diabetic rats. The protein level of hepatic PEPCK in diabetic rats was reversed by similar treatment with rhodiola-water extract to normal level and this action was also abolished by cyprodime (Figure 4B). The data for quantification of GLUT 4 and PEPCK protein levels were indicated in Figure 4A and Figure 4B, respectively.

## Discussion

In the present study, rhodiola-water extract lowers plasma glucose concurrently with an increase of plasma BER in STZ-diabetic rats. Both effects of rhodiola-water extract are dose-dependent in same dose ranges. STZ-diabetic rats are widely used as type-1 like diabetic animal model to investigate hyperglycemia [33]. Therefore, the plasma glucose-lowering and BER-elevating actions of rhodiola-water extract are concluded to occur under the lacking of insulin. When treated with rhodiola-water extract, STZ-diabetic rats provide a suitable model to explore the phenomena that the reduction of plasma glucose concentrations and increased BER are associated in diabetic rats.

Many actions of endogenous β-endorphin are recognized to be mediated by the opioid μ-receptors including the regulation of glucose homeostasis [34,35], but the mediation of opioid delta-receptor located in skeletal muscle regarding to the hypoglycemic effect of BER has also been demonstrated [36,37]. Actually, it has been indicated that the expression of β-endorphin sensitive receptor on skeletal muscle is vastly increased in type 1 and type 2 diabetic animals [38,39]. Moreover, the increase of hepatic opioid μ-receptors was related to diabetic hyperglycemia [40]. Thus, opioid μ-receptor blockade was employed to evaluate the involvement of this receptor in rhodiola-water extract-induced plasma glucose lowering action in STZ-diabetic rats. The blood glucose lowering effect of rhodiola-water extract is similar to meforminin in STZ-diabetic rats as described in previous reports [41,42]. Cyprodime, the selective opioid μ-receptor antagonist, has been shown to effectively abolish the activation of opioid μ-receptors [43,44]. The ability of rhodiola-water extract to decrease plasma glucose was actually suppressed by blockade of opioid μ-receptors with cyprodime. These findings demonstrate the activation of opioid μ-receptors through increased circulating β-endorphin in the plasma glucose lowering action of rhodiola-water extract in diabetic rats. Similar action was also observed in another herbal principle [45].

Endogenous opioids can be released into the bloodstream from glands other than the pituitary gland [46], and the secretion of opioids from the adrenal gland was observed to foster a reduction in plasma glucose in STZ-diabetic rats [28]. Thus, bilateral adrenalectomy was performed in the present study to verify that the source of the increased plasma BER observed in STZ-diabetic rats in response to rhodiola-water extract was the adrenal gland. As shown in Table 1, the plasma glucose lowering action and the increase of BER induced by rhodiola-water extract in STZ-diabetic rats were both attenuated by adrenalectomy. Thus, secretion of endogenous β-endorphin from adrenal gland is responsible for the plasma glucose lowering action of rhodiola-water extract in STZ-diabetic rats. Decrease of plasma glucose by β-endorphin has been mentioned in previous reports [47,48].

In diabetes, hyperglycemia is considered the consequence of increased hepatic glucose output and decreased peripheral glucose uptake [49,50]. Insulin deficiency is associated with changes in hepatic metabolism including increased expression of PEPCK that is a key enzyme of hepatic carbohydrate metabolism [49,51]. Additionally, decreased expression of skeletal muscle GLUT 4 in diabetes resulted in the reduction of insulin-mediated glucose uptake into skeletal muscle [52]. It was interesting to ascertain whether rhodiola-water extract produced plasma glucose lowering action in diabetic rats by overturning the diabetes dependent reduction of GLUT 4 expression and/or increase in PEPCK expression. To provide sample time for alterations in gene expression, STZ-diabetic rats received the repeated treatment rhodiola-water extract for 3 days. Under these conditions, the increase in hepatic PEPCK gene expression due to hyperglycemia was abolished by rhodiola-water extract. The decrease of GLUT 4 expression in skeletal muscle was also reversed by the same repeated treatment rhodiola-water extract. These findings suggest that rhodiola-water extract shows plasma glucose lowering action due to changes in the expressions of hepatic PEPCK and muscle GLUT 4 in an insulin-independent manner.

It has been indicated that endogenous β-endorphin via activation of opioid μ-receptors located in peripheral tissues was found to serve as a positive regulator of glucose utilization and a negative modulator of hepatic gluconeogenesis in the insulin-deficient state [20,53]. Opioid μ-receptors antagonist was therefore used to evaluate the involvement of opioid μ-receptors in the effects of rhodiola-water extract on metabolic gene expression in diabetic rats. Actions of rhodiola-water extract regarding the increase of GLUT 4 expression in skeletal muscle and decrease of PEPCK expression in liver were abolished in STZ-diabetic rats with the blockade of opioid μ-receptors using antagonist (Figure 3). Furthermore, elevation of GLUT 4 protein expression and suppression of PEPCK protein expression in STZ-diabetic rats by rhodiola-water extract were also blocked by the same antagonist named cyprodime (Figure 4).

It has been reported that PKC is involved in the rate-limiting step in GLUT4 mRNA expression [54,55]. Also, the PLC–PKC pathway is mediated in opioid μ-receptor activation [20,44]. Thus, this pathway is involved in the regulation of GLUT4 gene expression, although the detailed mechanism(s) need more investigations in the future. Our data also consistent with the report showing that opioid μ-receptor activation might act as a negative regulator to modify the gene expression of PEPCK in insulin-deficient state [19,56]. However, the mediation of PLC-PKC pathway in regulation of PEPCK gene expression is still unclear [57]. Thus, the signals from opioid μ-receptor activation for regulation of hepatic PEPCK gene expression during the absence of insulin need to clarify in the future. Taken together, the normalizations of hepatic PEPCK and muscle GLUT 4 expressions in STZ-diabetic rats by rhodiola-water extract are mainly mediated via an activation of opioid μ-receptors mainly through the released endogenous β-endorphin.

Many ingredients are mentioned in rhodiola [58] including glycosides, flavonoids, phenilpropanoids, and others [59]. Also, another plant of the same genus showed merit in reduction of plasma glucose in type-2 diabetic animals [60]. Thus, it is important to find out the effective ingredients and/or related metabolites in this action of rhodiola in the future.

## Conclusions

In conclusion, our results suggest that rhodiola-water extract may enhance the secretion of endogenous β-endorphin from adrenal gland of STZ-diabetic rats. The plasma glucose lowering action of rhodiola-water extract is mainly mediated by β-endorphin release through an activation of opioid μ-receptors to achieve the higher of GLUT 4 gene expression and/or the decrease of hepatic PEPCK gene expression. Therefore, these findings provide evidence in support of rhodiola-water extract as the agent for handling of hyperglycemia in diabetic disorders in rats. However, the blood glucose lowering effect of rhodiola and/or active ingredient(s) on human needs more investigations in the future.

## Competing interests

The authors declare that they have no competing interests.

## Authors’ contributions

CSN participated in the design of the study and drafted the manuscript. LJC carried out animal experiments, Western blotting analysis, qRT-PCR and performed the statistical analysis. HSN conceived of the study, and participated in its design and coordination and helped to analysis of statistical difference. All authors have read and approved the final manuscript.

## Pre-publication history

The pre-publication history for this paper can be accessed here:

http://www.biomedcentral.com/1472-6882/14/20/prepub
